# Early Intervention for Hearing-Impaired Children—From Policy to Practice: An Integrative Review

**DOI:** 10.3390/audiolres15010010

**Published:** 2025-01-24

**Authors:** Luisa Petrocchi-Bartal, Katijah Khoza-Shangase, Amisha Kanji

**Affiliations:** Department of Speech Pathology and Audiology, Faculty of Humanities, School of Human and Community Development, University of the Witwatersrand, Johannesburg 2050, South Africa; katijah.khoza-shangase@wits.ac.za (K.K.-S.); amisha.kanji@wits.ac.za (A.K.)

**Keywords:** hearing impairment, deaf, hard of hearing, early intervention, policy, practice

## Abstract

Background/Objectives: Globally, many countries have endorsed the World Health Organisation’s (WHO) early intervention (EI) guidelines through their legislation with contextual variations. Sensitive evaluation of gaps within EI-recommended systems is essential, especially in the translation of policy into practice across high-income and low- to middle-income country (LMIC) contexts, including South Africa. The main objective was to explore and identify the current evidence reflecting the application of hearing-specific government policy regarding EI and early education (EE) for hearing-impaired/d/Deaf/hard-of-hearing (HI/D/HH) children aged six and below. Method: An integrative review was conducted on peer-reviewed articles that examined policy in practice regarding EI for HI/D/HH children aged six and below. Studies were accessed via four databases (Ebscohost, Sabinet, Scopus, and ScienceDirect) and one search engine (Google Scholar) between 2014 and 2024. Qualitative evaluation ensued of themes identified deductively. Results: Twenty-six peer-reviewed studies were included. Deductive thematic analysis revealed six derived themes: EI timing, early hearing detection and intervention (EHDI)/EI mechanisms; EI services, EE, family considerations, and policy. Five of the 26 directly scrutinised government policy in its EI/EE practical application. Articles reflected the need for consideration of the complex processes that allow for policy actualisation, such as adequate infrastructure and family considerations. Conclusions: A bottom-up approach to policy actualisation, with grass-roots contextual considerations such as EI access and caregiver concerns, may improve policy application. Current findings have implications, particularly for LMIC contexts including South Africa, where EI and EE policy scrutiny regarding hearing impairment/deafness specificity is imperative for understanding its application alignment.

## 1. Introduction

Globally, 34 million children require intervention for disabling hearing impairment [1]. The well-documented consequences of hearing impairment in children include developmental sequelae affecting speech, language, cognition, education, emotional and social well-being, literacy, vocation, and financial outcomes [1,2]. Disability, defined by the World Health Organisation (WHO) incorporates hearing impairment as a sensory disability [3,4]. International policies, such as the United Nations Convention on the Rights of Persons with Disabilities (UNCRPD) and the United Nations Convention on the Rights of the Child (UNCRC), promote the health and participation of children with disabilities [5]. However, the diverse nature of disabilities requires specific approaches, and successful implementation of international frameworks depends on national legislative and policy contexts [1,5].

High-income country (HIC) contexts have long established robust legislation for early hearing detection and intervention (EHDI) and support services for children with hearing loss [6,7]. These measures often adhere to standards from bodies like the WHO, United Nations Children’s Fund (UNICEF), Joint Committee on Infant Hearing (JCIH), and the Family Centred Early Intervention for Deaf and Hard-of-Hearing Children (FCEI-DHH) international consensus panel [6,8]. These bodies provide principles and benchmarks for early intervention (EI), whilst global differences are acknowledged [8,9].

In the United States (US), a complex, tiered policy approach, informed by the JCIH, guides early intervention (EI) for hearing-impaired children, involving multiple sectors based on the child’s disability needs [7]. The EHDI program, mandated at the federal level, ensures comprehensive support for infants with hearing loss [9,10]. The JCIH guidelines, particularly its 2013 supplement, emphasise EI, family-centred care, and active family involvement, considering linguistic and cultural diversity, with families fostering language-rich environments and emotional support [11,12]. The updated 2024 principles include feedback from the deaf and hard-of-hearing (DHH) and family leaders, cultural viewpoints, and recent research [8]. The updated FCEI-DHH stresses power-sharing partnerships between parents and EI providers, calling for these principles to be embedded in legislation and guidelines [8]. This is central to low- to middle-income country (LMIC) contexts, where additional challenges may hinder EI implementation for the HI.

LMICs often face obstacles such as widespread poverty, limited government investment in healthcare [13], and a high burden of disease, making hearing impairment a lower priority [14]. A major challenge is the lack of specialised knowledge and capacity to provide necessary services for children with hearing impairment [13,15]. Social beliefs, stigma, and misunderstandings about ear and hearing disorders further hinder the goals set by the JCIH [9,12,13]. Due to these delays, EI in LMICs may extend to five years of age [16], unlike the birth-to-three-years window in HICs [9]. Early education (EE) for hearing-impaired children must thus be included in the EI paradigm in LMICs.

South Africa exemplifies the complexity of navigating policy in an LMIC context. Despite being one of Africa’s more advanced economies, it faces barriers and legislative gaps in addressing the needs of young children with hearing impairment [17,18]. Since 1994, South Africa has made notable advancements in early childhood development (ECD) policy, a key national priority. This is evident in documents like the 2030 National Development Plan [19], the Department of Social Development’s (DSD) ECD Integrated Programme of Action [20], and the National Integrated ECD Policy [21]. The importance of investing in ECD for academic and employment outcomes is underscored [22].

The crossover of policy between government entities like health, social development, and basic education has been challenging. The South Africa Department of Health (DoH) is responsible for early disability identification, rehabilitation, support services, and assistive devices, including those for hearing-impaired children [4,23]. The Health Professions Council of South Africa (HPCSA) provides guidelines on EI for the HI, emphasising family-centred EI for HI/D/HH children and EE [23]. Its EHDI guidelines advocate for intersectoral collaboration among health, social development, basic education, and the private sector [23]. The EI process should be family-centred and community-based, reflecting cultural congruency [23]. This mirrors JCIH and FCEI-D/HH recommendations, emphasising family partnerships with EI specialists like audiologists and educators [23]. In the quest to address South Africa’s many contextual challenges to ensure equitable, affordable, and targeted ECD services, intersectoral collaboration is recognised as key [24]. A scale-up approach to EI for the HI/D/HH is recommended with legislative support and private-public partnerships. The HPCSA [23] refers to intersectoral collaboration, but analysis of intersectoral interface detail remains scant, particularly regarding how individual government sectors are to integrate services for the child with HI.

Research has focused extensively on hearing screening and diagnosis both within HIC [25] and LMIC contexts [26,27,28] and diagnosis [29,30,31,32,33], and management outcomes [34,35]. However, relatively sparse information appears evident on EI, specifically in reference to policy in practice for hearing-impaired children. Specifically, a comprehensive understanding and practical application of hearing health care, encompassing intervention and holistic management post-hearing device fitting, within the context of EI and EE policies and their practical application, is required. Tailoring policy design for contextual relevance, as delineated above, is essential. Given the additional complexity often experienced by LMICs, EI processes within these developing contexts are often not formally supported at government level. Within the South African context specifically, EI processes for the HI are not formally mandated by South African law, despite HPCSA involvement. Thus, the first imperative, as part of a larger research effort, is to identify and describe the gaps in hearing impairment/d/Deaf/hard-of-hearing policy in its application for children aged six and below in HIC and LMIC contexts, including South Africa.

## 2. Materials and Methods

For a consolidative review on “EI for the HI—From Policy to Practice” in the South African context, a robust review methodology that ensured comprehensive coverage and critical analysis of available evidence was adopted. To identify and describe current gaps in hearing impairment/d/Deaf/hard-of-hearing government policy and influencing guideline application for children aged six and below, an integrative review of the literature was conducted. The sources included empirical and theoretical peer-reviewed articles globally.

### 2.1. Search Strategy

To maintain study rigour, systematic review principles regarding database and search engine searches were employed [36]. These included searching the online databases Ebscohost, Sabinet, ScienceDirect, Scopus, and the search engine Google Scholar. The specific keywords that were applied included the following: early intervention, early education, hearing impairment, deaf, hard-of-hearing, policy, guidelines, and services. Boolean operators such as “AND”, “OR”, asterisks, and parentheses were used where appropriate to refine source articles detected. To ensure current data within a more present-day focus, a timeframe relative to JCIH, FCEI-DHH, and HPCSA guidelines was chosen between January 2014 and May 2024. In addition, rigorous perusal of relevant articles’ reference lists was conducted to identify further sources. The primary researcher sourced all articles, and an iterative process was applied to refine the final selection. All articles initially selected and those included in the final dataset were reviewed by two secondary researchers to ensure the appropriateness of the final article selection.

### 2.2. Inclusion/Exclusion Criteria

**Figure 1:**
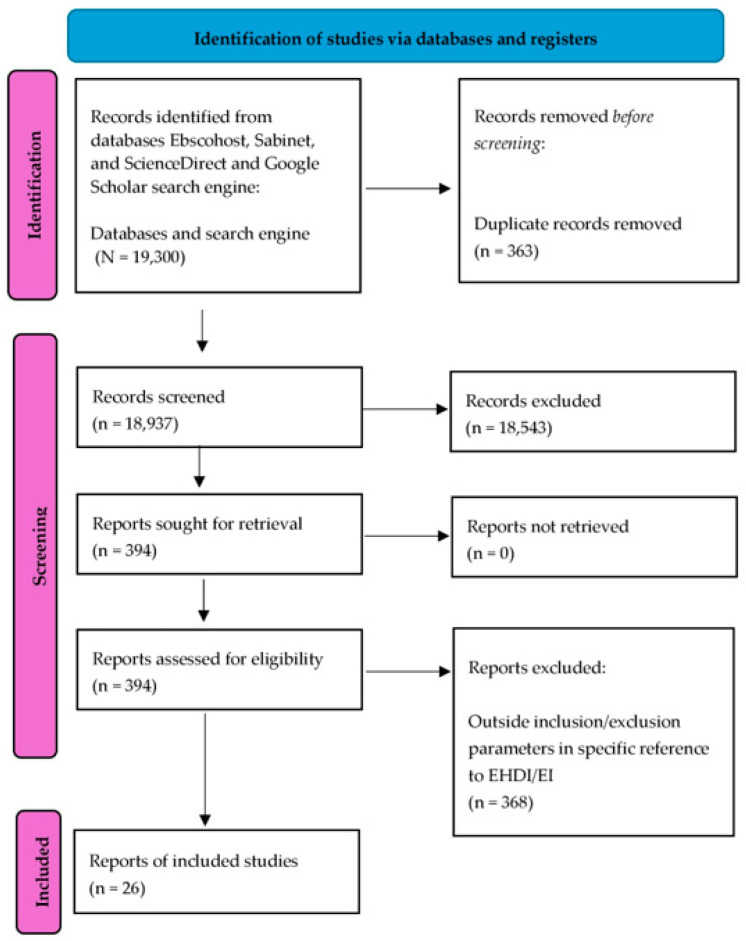
PRISMA-ScR flow diagram—PRISMA model approach [36].

Articles were required to be of primary research, reflected as empirical or theoretical research. Narrative, systematic, and integrative reviews were thus excluded. The articles were required to be published in peer-reviewed journals, and available in English to align with the researchers’ language skills. Grey literature was thus excluded, particularly to increase reliability and validity by ensuring research quality and to contain diverse formats. The focus was on hearing impairment/d/Deaf services and influencing factors in reference to government policy and/or guidelines promulgated by relevant consensus statements (JCIH; FCEI-DHH) and/or working groups (HPCSA), from which government entities often derive input. Figure 1 depicts the search strategy and the results obtained.

### 2.3. Data Extraction and Synthesis

The review period spanned January 2014 to early May 2024. Across databases, a total of 19,300 articles were detected in reference to the key words with Boolean operators applied. Of these, through a linear, progressive process as depicted in Figure 1 above, 26 journal articles were identified as appropriate for final inclusion. The emphasis was on government policy and/or influencing guideline consideration, including factors directly related to policy implementation such as logistics. Deductive thematic analysis was applied through the application of Braun and Clarke’s (2006) six steps to thematic analysis, namely, data familiarisation, code generation, theme derivation, theme review, theme defining and naming, and report generation [37]. Themes were validated by reviewing themes, clearly defining themes with refinement and continued analysis, and researcher reflexivity [37]. Specifically, initial codes were generated by the first author highlighting relevant text segments in article hard copies. Codes were then reviewed, patterns identified, and connected codes collapsed into relevant themes. Discrepancies were discussed amongst authors, and, if necessary, themes were modified.

## 3. Results and Discussion

For better cohesion, integrated expression, and concept clarity, results and discussion are included under one section.

### 3.1. Overview of Included Studies

The 26 articles included twenty empirical studies (quantitative, qualitative, and mixed methods designs) and six theoretical studies and were heterogeneous in nature (depicted in table format in Supplement Table S1. Extraction included context and location, method, results inclusive of themes derived, and recommendations. Thirteen articles were located within HIC contexts and 13 within LMIC contexts, with South Africa contributing 10 within the LMIC milieu.

On application of the deductive thematic analysis approach [37], more than one theme was derived for six of the 26 articles, with percentages per theme calculated out of the total number of times the themes were evident (*n* = 30). The six derived themes include EI Timing (*n* = 4; 13.3%), EHDI/EI Mechanisms (*n* = 8; 26.7%), EI Services (*n* = 3; 10%), EE (*n* = 3; 10%), Family Considerations (*n* = 3, 10%), and Policy (*n* = 9; 30%). Detailed results are included in Supplement Table S1. Studies are discussed per theme below according to their predominant emphasis and expression. Of specific note, is that even though nine articles were included under the theme of Policy, only five studies directly scrutinised government policy in its practical application. These included Deng et al. (2020), Alam et al. (2016), Curle et al. (2017), Oppong et al. (2023), and Ndegwa et al. (2024) [38,39,40,41,42]. The six major themes, with sub-themes, are presented and discussed below, revealing their inter-relatedness through specific details and recommendations provided by the respective authors, along with relevant implications.

### 3.2. Themes

#### 3.2.1. Theme 1: EI Timing (*n* = 4; 13.3%)

The scope of EI Timing within this review was defined as articles with a specific focus on JCIH (HIC EI for HI/D/HH children by age six months, preferably by age three months) or HPCSA (SA LMIC by maximum 8 months) EHDI timing recommendations for better childhood outcomes [9,12,23].

The importance of these EHDI/EI timelines was thus considered an appropriate starting point for the evaluation of guidelines and policy application in the field of hearing impairment/d/Deafness, as EI can only follow early identification of hearing loss. To provide insight regarding the knock-on effects that impinge on EI, diagnosis and hearing aid fitting timelines are detailed below.

Specifically, this review includes four studies that evaluated compliance with EHDI/EI timelines, where objectives included evaluation of alignment with either JCIH 1:3:6 [9,12] or HPCSA maximum 1:4:8 [23] guidelines and associated variables. Awad et al. [43] focused exclusively on this area within the HIC context of a metropolitan area, (Colorado, US), with their results reflective of timely hearing aid fitting of infants by three (14%) or four months of age (48%), with an EI average enrolment age of less than six months. The barriers to timely fitting included appointments missed/cancelled, middle ear involvement, and degree of hearing loss, specifically mild hearing loss. Better alignment with JCIH guidelines was felt to be required through adjustments in scheduling and appointment options, e.g., reducing the number of appointments through combining visits, minimising identification and hearing aid fitting age, and enhancing stakeholder communication and parent education. Recommendations included future studies on family barriers, service provision alternatives (e.g., telehealth, travelling audiologist), socioeconomic status impact and impact of additional medical needs, and family stress level evaluation.

Similarly, in the LMIC context of Saudi Arabia, Alyami et al. [44] investigated EHDI/EI timelines. Results revealed a markedly delayed diagnosis, hearing aid fitting and EI service commencement mean age, well beyond the JCIH recommended benchmarks. Participants outside Riyadh received later interventions compared to those in Riyadh. Barriers were considered to be EI accessibility issues (geographical, financial, and infrastructure-related), which emphasises the role EHDI mechanisms play in EHDI’s success. These aspects are explored in more depth under the theme EHDI Mechanisms below.

Within the LMIC context of South Africa, Meyer et al. [45] aimed to determine the average age at diagnosis and hearing aid fitting, and the time lapse between diagnosis and intervention nationally within the private hospital sector. It must be noted that although South Africa is contextualised as an LMIC country, the private healthcare sector is regarded as resource-intensive and highly developed [46].

Their finding revealed a markedly delayed diagnosis, hearing aid fitting, and EI service commencement mean age, well beyond HPCSA-recommended timeframes [23]. Despite the lag between diagnosis and hearing aid fitting being commensurate with international JCIH benchmarks, the general consensus was that the delays regarding diagnosis, hearing aid fitting, and intervention were significant with a lack of adherence to HPCSA guidelines [47]. Contributing factors for delayed intervention were attributed to the family’s financial status, reliance on approval for medical aid funding and parental delays in return for follow-up appointments, denial, and seeking second opinions, as well as poor follow-up. These factors are often shared by LMIC contexts, where EHDI processes may be delayed for similar reasons [48,49]. A need for upscaling comprehensive and integrated EHDI services with a national database was emphasised, with the resolution of the identified administrative and financial barriers and reduced local options with referring out as the alternative.

In contrast to the private hospital context above, Khoza-Shangase and Michal [50] investigated EI within the South African public sector in Gauteng Province. Here, marked delays in hearing impairment identification, amplification provision and aural rehabilitation were noted in comparison to HPCSA recommended guidelines [23], with aural rehabilitation having commenced at a mean age of 2 years 5 months. Similarly, Maluleke et al. [34] also reported marked delays in HI identification and EI within the context of two EI preschools. Despite the appropriate provision of audiological interventions post-identification of hearing loss, the above reflects delays that significantly exceed recommended contextual HPCSA guidelines for early hearing loss identification and intervention [23]. To counter this, mandated newborn hearing screening is recommended to facilitate earlier diagnosis and intervention.

Although EHDI is often mandatory in HIC contexts such as the US [7], difficulties are experienced despite the broader HIC context. The heterogeneity of individuals and populations within individual countries necessitates contextual considerations especially when formulating and applying policy. HIC contexts thus also experience barriers to EHDI such as family barriers, parent education, and stakeholder communication. Such issues may be shared by LMIC country contexts depending on country-specific contextual factors such as poverty, burden of disease, and linguistic diversity. The South African context shares many of these aspects regarded as barriers to EHDI actualisation, and these factors must be duly considered in any policy formulation. The HPCSA [23] has done so in its well-considered EHDI position statement, but as these are not mandatory, a lag in meeting its 1:4:8 benchmarks is evident. Implications are raised for motivating EHDI policy formulation and implementation at a government level.

#### 3.2.2. Theme 2: EHDI/EI Mechanisms (*n* = 8; 26.7%)

The scope of EHDI/EI Mechanisms within this review was defined as EHDI/EI processes that impact EHDI/EI implementation from a practical perspective, including logistics, infrastructure, data management, geography as it impacts access, financial barriers [9], and service system capacity [23]. These EHDI/EI mechanisms either facilitate or hinder policy in its practical application. Nine studies raised, as a focus area, EHDI/EI mechanism factors that impinge directly on the success of EHDI/EI implementation. They are expressed accordingly as sub-themes as per the specifics delineated.

##### Sub-Theme: Data Management/Systems

Alam et al. [39] highlighted the lack of US data reporting standardisation with inconsistent and/or incomplete data relating to the 1:3:6 JCIH benchmarks. This is despite JCIH’s emphasis on the critical role standardised reporting plays for EHDI realisation at a federal level [9,12,51]. Alam et al. [39] emphasised that EHDI programs need to be directly linked to EI programs, but this may not occur if data sharing agreements are not in place and if jurisdictional privacy laws disallow data sharing. In addition, information systems may vary in capacity, in their definitions and programming. Alam et al. [39] reflected that the Centre for Disease Control (CDC), as of 2016, had attempted to remedy data management issues and standards issues for better data exchange and had shared data interpreting having made strides in improving surveillance data utilisation and assessment of EHDI process performance. Alam et al. [39] recommended that continued collaboration and commitment between the CDC, providers, other stakeholders, and EHDI programs is necessary to promote and strengthen EHDI information systems.

Holzinger et al. [52] demonstrated agreement with the positive influence secure data management wields over the EHDI and the EI enrolment process. In the HIC context of Austria, their quasi-mandatory, jointly HIC standard operating tracking procedure, under the guidance of health authorities, facilitated a reduction in the mean age of EI enrolment to within JCIH benchmark parameters of six months and under. They were able to do so within national legal parameters at no additional cost.

Similarly, Folger, et al. [53] evaluated statewide program effectiveness through their database on EI and educational outcomes for HI/D/HH children within the HIC context of Ohio’s state-wide collaborative. They concluded that linked databases could provide valuable information to enhance understanding of academic success predictors early in the academic process. Such resource development is useful to evaluate EI early enrollment effectiveness and to improve language and early academic outcomes, such as preschool and pe-literacy readiness. The interface between EI and EE systems is valuable in facilitating transition, whether at a database or outcomes level. Folger et al. [53] demonstrated that multi-agency collaboration and data integration are essential for supporting policy formulation and for evaluating and improving outcomes for children who are HI/D/HH.

Deng et al. [38], in their theoretical paper on EHDI in the US, reflected further on the importance of data management in the EI process, stating that US state and federal law authorise the CDC to provide technical support and funding to facilitate EHDI data tracking and management systems to ultimately achieve EHDI goals. Service provision was nonetheless sited to be influenced by racial, geographic, and socio-economic factors, which are acknowledged to be influencing factors in the EHDI actualisation process [9,12,23,47,51].

In contrast, Moodley and Störbeck [54] revealed that within South Africa’s LMIC context, there is no national data management system in use, nor is there a private or public consistent shared data system. Their findings revealed that within public and private sector agencies, either paper-based systems, a combination of paper- and computer-based systems, computer-based systems, or web-based systems are utilised, with paper-based systems predominating with usage by 44%. Electronic system usage, particularly within healthcare, has demonstrated greater accuracy and completion of documentation with useful indicators for policy planning and formulation [55,56]. Lack of staff and time were reasons given for reduced data entry which are challenges reflected internationally [54]. South African context-specific challenges, which are often shared with LMIC contexts, were cited as reduced access to electricity and internet connectivity [54] resulting in a lack of data-sharing capabilities.

##### Sub-Theme: EI Logistics and Infrastructure

Relatedly, Khoza-Shangase’s [16] study expanded on South African contextual challenges that compromise EI service delivery. These comprised logistical barriers including long travel distances to access services, service affordability, and limited accessibility and availability of suitable schools and health care services. This was confirmed in Maluleke, Khoza-Shangase, and Kanji’s study [57], where EHDI services failed to meet caregiver accessibility expectations resulting from long travel distances and associated high costs. As stated by Khoza-Shangase [16], these logistical challenges are recognised within the South African context, where socio-economic diversity and inequity continue to be a significant barrier, and constrained resources impede access to health and education services for children [58,59,60,61,62]. Khoza-Shangase [16] posits the need for careful consideration of logistical and infrastructure barriers to service delivery in policy formulation for EI services to be successful.

Similarly, Naidoo and Khan [63], in their study on EHDI barriers and facilitators in the South African province of Kwazulu-Natal, identified barriers as cited by audiologists/speech therapists in private and public healthcare facilities, to be reduced healthcare resources in private and public healthcare facilities, inclusive of staff shortages. Some staff were cited to have had poor EHDI awareness and knowledge. Recommendations for improvement included the need for context-specific solutions to improved resource allocation through resource and infrastructure investment inclusive of data management, better human resource allocation and EHDI training, and enhanced collaboration among healthcare professionals with intersectoral collaboration. Their recommendation to mandate EHDI would assist in facilitating these processes, where formal policy formulation and planning at government level would need to consider EHDI/EI mechanisms such as logistics, budget allocation, human resource planning, and sector networks within health care and education. Naidoo and Khan [63] posit tele-audiology as a consideration in EHDI service delivery especially in resource and staff-restricted contexts. They elaborate that the implementation of policy faces significant challenges due to resource limitations. Barriers to EHDI stem from financial constraints affecting government and healthcare facilities [63]. It is well documented that these constraints impact staff employment, equipment provision, and access to hearing aids in public healthcare settings [14,58,63,64]. The human resource issue is also well documented by the current authors, with Kanji specifically reporting human resource issues to be a threat to the South African National Health System [59,65].

##### Sub-Theme: Tele-Audiology

Naidoo and Khan [63], as previously mentioned, proposed tele-audiology as an additional service delivery method to the EHDI process to facilitate access, especially in LMIC contexts. Surprisingly, Cole et al. [66] reported limited telehealth adoption in the HIC contexts, such as Colorado, US. This is despite its reported flexibility, which enables additional visits and sessions outside traditional hours and where families in rural areas can gain access to specialists through telehealth. Cole et al. [66] revealed that despite the telehealth model’s ability to enhance family engagement and coaching practices, its challenges included web technology issues, negative family attitudes, and insufficient provider training in family coaching within their context. In addition, their findings revealed that some providers and service coordinators perceived telehealth as less effective and less personal. The current researchers posit that telehealth is an avenue that requires further investigation, particularly in the post-COVID-19 era and in contexts where service access proves challenging. The JCIH [9] and the HPCSA [23] support tele-intervention as viable options for EI with specific contextual consideration. Again, policy planning at higher government levels would need to unpack viability and population support, especially for families in financial hardship.

### 3.3. Theme 3: EI Services (n = 3; 10.3%)

The scope of EI Services within this review was defined as EI treatment intervention types and intervention intensity post-diagnosis, including hearing aid fitting, FCEI-DHH, and aural rehabilitation. Three studies were identified that specifically raised EHDI/EI treatment factors that impinge directly on the success of EHDI/EI implementation. They are expressed accordingly as sub-themes as per the specifics delineated.

#### 3.3.1. Sub-Theme: EI Service Type

As a precursor to programme intensity, EI service selection requires perusal. Meyer et al. [45] detailed private service provision of EI services to HI children as providing some form of EI services to the majority of their private client base inclusive of hearing aid fittings, where the majority of the latter occurred by a maximum of 36-month EI. They described EI services to have included parent counselling, parent guidance, speech-language intervention, support group services, and auditory training. Of the participants, referrals to outside interventionists predominated, with referrals of children to other private practices, schools or intervention centres non-governmental organisations such as Hi Hopes [67], and public hospitals when their services or the patient’s funding fell short. The current authors agree with Meyer et al.’s conclusion that upscaling South African EHDI service infrastructure, as mentioned under EHDI/EI Mechanisms, will improve the integrity and availability of EI services. This would require upstream policy planning with input from service providers to sector policymakers.

Khoza-Shangase and Michal [50], as part of their objectives, aimed to investigate audiological management protocols, amplification provision, and mode of communication at three state hospitals in the South African public sector. Their findings revealed that all children were aided appropriately; however, considerable delays were noted regarding aural rehabilitation introduction mean age, with aural rehabilitation approaches provided to 85.71% of children having included auditory verbal therapy, sign language, and total communication. Even though HPCSA [23,47] EI benchmarks were not achieved, when EI was provided, it was appropriate [50]. Reasons expounded were possibly attributable to reduced parental knowledge regarding hearing impairment, reduced staff–patient ratios, and patient and staff burden of disease priorities. Improved EHDI/EI mechanisms such as funding and infrastructure with a mandated EHDI platform at the government level can assist in improving EI services and outcomes. Störbeck [67] argues that ECD investment should encompass financial support, allocation of resources, expertise, and dedicated time. The execution of these initiatives should be tactically planned and culturally aware, accompanied by public declarations and responsibility with government imperatives to acknowledge and uphold the human rights of children with developmental disabilities.

#### 3.3.2. Sub-Theme: EI Service Intensity

EI service intensity is often driven by developmental needs. Service intensity has been shown to improve language outcomes in preschool, although Geers et al. [68] report studies to be scarce in terms of evaluating intensity measures. As such, Meinzen-Derr et al. [69] investigated the EI intensity-associated factors within IDEA Part C. They found that most Ohio children received four or more EI services, inclusive of service coordination (for individualised service plans) and specialised DHH services, special instruction, speech-language therapy, and family training. Service intensity nearly doubled from the first individualised family service plan (IFSP) in the first year. Although service intensity varied across regions, higher EI intensity was associated with prematurity, risk indicators for hearing loss, severe/profound hearing loss, bilateral hearing loss, and disability. Children with bilateral hearing loss, severe/profound hearing loss, and developmental disabilities received more intensive therapy. Enrolling in EI by 6 months was linked to lower intensity compared to enrolling after 12 months. African American children received significantly lower EI intensity than white children. This is especially relevant in the South African context, where access to healthcare services is hindered by an overloaded system, inequalities, scarcity of healthcare professionals, language barriers, and cultural diversity; these challenges disproportionately impact vulnerable individuals in rural and economically disadvantaged black communities [70]. The current authors support Meinzen-Derr’s [69] assertion that grasping the extent of EI service intensity, correlated to its results for young children with hearing impairment, can in turn facilitate understanding of where public EI government-backed programmes should direct essential resources in EI service delivery to enable HI children to flourish.

### 3.4. Theme 4: EE (n = 3; 10.3%)

As a component of ECD, early childhood education is inextricably linked to a child’s developmental outcomes as a follow-on or part of EI, where a child’s multidisciplinary team may include educators [67]. Three studies were identified for inclusion in this review under the theme EE, where transition and school outcomes were an area of focus considered to affect policy formulation and development.

Curle et al. [71] aimed to investigate communication among parents, teachers, and providers in EI during the HI/D/HH child’s transition to preschool. Findings revealed that clear communication with high-intensity support during the transitional process facilitated the route parents needed to navigate with their HI/D/HH child. Parents appreciated the trusted relationships and prompt responses with their early interventionists. Comprehensive information and resources from EI programs proved beneficial. Information sharing and emotional support provided by early interventionists assisted in addressing parental anxiety. Early interventionist written reports to teachers of the deaf (TOD) were considered essential for preparing necessary accommodations. However, the lack of established communication pathways between schools and EI systems hampered the transfer of knowledge and information. Once their child entered school, parents preferred regular teacher updates, and communication with more experienced parents served as a valuable source of support. Communication between stakeholders is significant in ensuring smooth transition pathways for HI/D/HH children as they move from EI to EE. Reciprocal programme communication must be considered at a policy level to ensure implementation.

Likewise, Zaidman-Zait et al. [72] aimed to explore transition support practices and parents’ concerns as well as teacher and parent perspectives regarding supportive practices that facilitate smooth school transitions for DHH children. Key parental concerns included their child’s participation abilities in inclusive settings and the support they would receive. Teachers reported important transition practices to include parent–school communications and determining suitable educational placements. Differences in perspectives between parents and teachers were noted, particularly regarding advocacy and family resources. Unformalised transition processes may result in variations across preschools in the way HI/D/HH children are transitioned into EE. As such, more formalised policy protocols may ensure standardised processes, which may assist in allaying parental anxieties.

Within the LMIC South African context, Maluleke et al. [34], aimed to investigate preschool children’s communication and school readiness abilities in EI preschools for the HI/D/HH. Their findings revealed that only three of eight participants achieved age-appropriate school readiness because of late identification of hearing impairment with late EI initiation. School readiness criteria included communication abilities, attention/listening skills, early literacy skills, and mathematical concept knowledge. They concluded that swift commencement of EI services is vital for improved communication and preparedness for school. They further expressed that the urgent execution of extensive EI services is essential to achieve opportunity parity for children with hearing impairments. These results further suggest the need for methodical planning and execution of globally recognised best practices at different stages of service provision, within both the Health Department and the Basic Education Department. By identifying and starting hearing impairment intervention services early, improved communication and school readiness may be facilitated with possible developmental parity when compared with children who do not experience hearing impairment [34].

### 3.5. Theme 5: Family Considerations (n = 3; 10.3%)

The scope of Family Considerations within this review was defined as family needs as the priority focus as expressed or anticipated by caregivers and providers. This review included four studies in this regard.

Ward et al. [73] aimed to investigate the family needs of young HI/D/HH children as expressed by parents, EHDI coordinators, and EHDI-contracted family-based organisations (FBO). Results revealed the family need for comprehensive information at different stages within the EHDI process, with trusted culturally congruent support with resources that are well coordinated. Inter-family support was also expressed as useful. Additionally, the need for reliable, easily navigated websites and the value of having a single contact person to help connect families with resources were highlighted. It was felt that consistent information from service providers to parents improved outcomes with a focus on language, socio-emotional development and literacy. Stronger EHDI–FBO partnerships are required with FBO technical assistance and support. Family leadership barriers were reported to include available time, cultural challenges and confidence. Despite the HIC world context, cultural factors are always at play given the increased population heterogeneity as a function of immigration [74]. In this HIC context, Ward et al. [73] reported that 25% of EHDI funding, as per the Federal Maternal Child and Health Bureau, is apportioned to FBOs, which demonstrates forward thinking in the EHDI process. The complexity of broad-reaching, tiered planning at government level is underscored and is recommended for LMIC contexts such as South Africa. In LMIC contexts, governments may leverage existent structures such as primary healthcare and may further incentivise daycare facilities to become more formalised with compliant EHDI services offered. Family leadership cannot be underestimated especially as the main driver in the EI process. FCEI-DHH constructs and guidelines emphasise the family role as key in the EI process [8,9,23], and it is families who have confirmed the desire to lead, as demonstrated by Ward et al. [73].

Maluleke [75], specifically within the South African context, emphasises the importance of incorporating family members of children who have been diagnosed with hearing impairment as equal partners in EHDI programmes, with due consideration of their cultural and linguistic needs. Optimising developmental outcomes may be achieved through empowering caregivers through cultural and linguistic congruency, which further encourages the child to identify with their cultural and linguistic heritage [23,76,77]. In addition, family-centric EHDI programmes would stem language barriers caregivers experience in EHDI access [16,57,76,77]. The HPCSA [23], as an arm of the South African DoH, expounds family centricity within its EHDI guidelines, but further steps are necessary for upstream policymaking to actualise EI processes where family-provider partnerships are at the EI core.

In further reference to the South African context, Maluleke et al. [57] report that caregivers expected timely hearing impairment diagnosis, but this only occurred for two of the 11 children, as the parents had insisted on hearing evaluation at their birth. Most participants expected easier EHDI access and expressed EHDI access frustrations with long distances to EHDI services, high service costs, and language barriers, with English spoken throughout. In addition, the rarely achieved age-appropriate spoken language acquisition was an essentially unmet expectation in their study’s nine of the 11 children due to delayed EHDI service provision. Mainstream education enrolment was another of the study’s mostly unmet parent expectations, where only one out of 11 children was enrolled in a mainstream school. Integrated, interdisciplinary, and contextualised EHDI services are needed to cater to South Africa’s diverse population, especially if the HPCSA’s (2018) EI recommendations are to be met. Understanding caregiver expectations and incorporating caregiver involvement are crucial for successful EHDI services [57]. As cited by Maluleke et al. [57], relinquishing ECD initiatives translates into compromised community well-being, with poverty cycles prolonged, socio-economic suppression and inequality perpetuated, and educational improvement denied [78]. Störbeck [67] argues for the defence of children’s rights to FCEI, specifically in the global South, through the HI HOPES evidence base, a supportive FCEI programme catering to the needs of HI/D/HH children up to age three and extended to age six where necessary. Störbeck [67] highlights the home-based Hi-HOPES early childhood intervention (ECI) approach as a multidisciplinary, comprehensive model that supports families with young children who are HI/D/HH in an empowering partnership. This enables informed family decision-making without language or communication approach bias. In this way, optimal child development may be fostered in conjunction with improved caregiver confidence and skills required for advocating for their child’s well-being and rights. Störbeck [67] cites HI HOPES as a powerful example of the transformative influence family-centred ECI can wield in empowering families and encouraging inclusive education. However, funding issues prevail, and as such, Störbeck underscores the imperative for stakeholders and national governments to prioritise initiatives in ECD.

### 3.6. Theme 6: Policy (n = 9; 30%)

The scope of Policy within this review was defined as policy evaluation and commentary as a focal point. This review thus included eight articles that did so. Deng, Gaffney, and Grosse [38] in their review of EHDI state services, highlighted public health infrastructure development to ensure EHDI provision as well as the challenges experienced. In the US, EHDI federal funding is apportioned through the CDC and the Health Resources and Services Administration (HRSA). Deng et al. [38] commented that state funding is crucial for effective EHDI services such as data management and information systems, and the CDC and HRSA were and are instrumental in facilitating integrated EHDI information systems. Even in the US HIC context, challenges as recent as 2016/2017 in meeting EHDI/EI benchmark timelines have prevailed possibly in part due to state statute differences, with state variations in policies and regulations and state differences in infrastructure and capabilities. Interestingly, Deng et al. [38] reported that, despite the HIC context milieu, as of 2020, reliance on old technologies still exists within some US states. In addition, they highlighted that data definition variations in EHDI state programs have hindered CDC monitoring, as previously confirmed by Alam et al. [39] and Mason et al. [79]. The reported geographic, socio-economic, and racial disparities that were observed despite the HIC world context, as previously cited by Bush et al. [80], Deng and Finitzo [81], and Lantos et al. [82], underscore the urgency for collaborative efforts by government entities and other stakeholders.

Curle et al. [40] evaluated the transition of the HI/D/HH child from EI to school specifically in reference to procedures, organisational policies, and guidelines. Programme administrators were recruited as participants, where facilitators and barriers to smooth transitioning between EI and EE were identified. Results revealed five categories of smooth transitioning facilitators identified by administrators.

Specifically, many participants highlighted the significance of effective communication, including document exchange, among stakeholders, while the lack thereof was viewed as a barrier to the transition to preschool. Despite efforts by EI systems to educate parents, misinformation regarding the school system’s transition process and available resources persisted. The Curle et al. [40] study highlights the importance of clear communication in facilitating the transition process and preventing misunderstandings. Successful relationships between stakeholders, such as educators, parents, and service providers, are fostered through fluid communication and regular interactions, as reinforced by Rimm-Kaufman and Pianta [83]. Rimm-Kaufman and Pianta [83] affirmed that interactive patterns between institutions, groups, and individuals significantly affect transitions and educational outcomes for children. This underscores the recognised importance of clear communication among stakeholders with clear policy directives as drivers. In addition, time was also identified as a significant factor in the transition, where allowing for adequate preparation and collaboration among stakeholders is key [40].

Barrier-wise, a lack of knowledge and resources posed barriers to the smooth transition of children with disabilities and hearing loss to preschool settings. Curle et al. [40] commented on the lack of awareness among school administrators and teachers regarding the needs of HI/D/HH students, as previously noted by Rude et al. [84] and Marschark et al. [85], leading to disagreements in educational placements. Additionally, Curle et al. [40] also noted the diminishing availability of specialised programmes and educators for HI/D/HH children, as already highlighted by Moores [86] and Dolman [87], which further challenges the transition process. Financial constraints also impacted the transition from EI to EE, particularly for HI/D/HH children. The high costs associated with specialised accommodations and services, such as amplification equipment and sign language interpreters, were reported to strain school district resources, as already highlighted by Chambers et al. [88]. Curle et al. [40] highlighted that the financial barrier underscored the need for advocacy at both the local and governmental levels to ensure adequate support for HI/D/HH students. They further asserted that decisions made by school districts, influenced by government funding choices, directly affected the support available for HI/D/HH students. Lastly, the lack of information or misinformation about school systems that was evident amongst EI personnel pointed to a disconnect between EI programs and school districts, highlighting the need for better collaboration and communication, as already asserted by Kagan and Kauerz [89]. Improving coordination and information-sharing among stakeholders is essential for enhancing the transition process from EI to school for children with disabilities inclusive of hearing loss. Strengthening planning efforts and communication channels can help address these challenges and facilitate a more seamless transition for these children [89]. Overall, addressing these knowledge gaps, resource limitations, and financial challenges is crucial in promoting a seamless transition for children with disabilities from EI to EE settings. Integrative policy is key to preventing the fragmentation of systems, which influences the transition from EI to EE.

Similarly, Davenport and Weir [90] acknowledged the complexity of navigating the transition from EI to EE, where the process takes place at 2.5 years as part of the move from IDEA Part C to IDEA Part B. Davenport et al. [91] acknowledged administrator challenges as those highlighted by Curle et al. [40] and posit the TEAM approach to assist with this transition process in alleviating many of the challenges highlighted by Curle et al. [40]. Their theoretical article [90] expounds on this approach where “T” stands for Transition Planning, “E” stands for Educational Team, “A” for accommodations, and “M” for Making Connections. The key components of the TEAM approach include the following:

- Transition Planning: Helping families prepare for the transition process, including facilitating understanding of the differences between EI and preschool services (Part C and B, respectively), as the child approaches 2.5 years old.
- Establishing an Educational Team: Collaboration between parents and professionals, such as general and special education teachers, administrators, and service providers, to ensure effective child support.
- Providing Accommodations: Identifying and implementing appropriate accommodations to support the child’s social interactions, learning, and communication, such as communication strategies, assistive technology, and individualised education plans.
- Making Connections for Continued Success: Emphasising continuous communication and collaboration among educators, parents, and service providers to meet the child’s educational needs. Of particular interest are the connections referred to as in-house specialists and state and national organisations, which are personnel resources aimed to provide all-rounded comprehensive support in the form of expertise to guide the transition process.

By adhering to these principles, the TEAM approach aims to facilitate a smooth transition for HI/D/HH children, addressing challenges related to communication, advocacy, and fostering positive peer relationships. Ultimately though, despite IDEA Part C’s and Part B’s alignment, further resolution is required regarding fragmented policy processes in its intricacies.

Snoddon [91] reported on fragmented and sometimes informal services in Ontario’s (Canada) early childhood education and care system. Snoddon’s [91] narrative case study from three participant reflections located in a daycare setting, revealed language, funding, infrastructure, and policy issues. Specifically, Ontario’s early childhood education and care (ECEC) setting, having moved to an inclusive education model, is reported to not adequately address the needs of deaf children benefiting from sign language. Government support for spoken language development appeared to outweigh that for signed language development, favouring the former and parents frequently are reportedly required to pay out-of-pocket for American Sign Language (ASL) services due to these gaps. The policy requiring parents to choose between auditory–verbal therapy (AVT) and sign language services, rather than promoting bilingualism, reportedly posed challenges as did the design of publicly funded EI sign language services which often appeared to clash with the ethos of inclusive ECEC systems. These ASL issues are of significant concern, where parental autonomy regarding language choice for their child is curtailed. Furthermore, Snoddon [91] reported deaf early childhood educators faced a shortage of qualified personnel and training opportunities. In addition, the then-recent policy changes in the Ministry of Education’s preschool home visiting program were reported to have reduced flexibility and support available. Such policy barriers and limited access to resources in the HIC Canadian context appear to have resulted in significant gaps in public services with service fragmentation for HI/D/HH children. In addition, policy changes appear to have restricted the delivery of EI services, leading to the breakdown of previously integrated services. Snoddon [91] reported a need for more training and collaboration with deaf professionals in ECEC and EI, where creating bilingual ASL environments within ECEC settings is considered essential. A point was made for better leadership from daycare management and government policymakers. As such, Snoddon [91] concludes that inclusive ECEC for deaf children, at present, appears to be an unrealised aspiration.

In reference to LMIC contexts, rather than the above HIC contexts, Oppong, Swanwick, and Fobi [41] recently examined Ghana’s inclusive education policies and practices for young deaf children. A focal point of investigation as they relate to Ghanaian policy was the connection points between parents, clinicians, and teachers, as well as the clinician and educator precarity and resources available to sustain inclusive education. Findings from their clinician and educator interviews revealed that parent–teacher association (PTA) meetings serve the purpose of educating parents about basic sign language and offering counselling. In addition, challenges faced included parents’ restricted weekend visits, financial limitations, and infrequent PTA gatherings. Excursions and interactions with mainstream schools were reported to contribute to social and communicative development. Clinicians emphasised showcasing successful deaf individuals and providing emotional support. The study findings highlighted a discrepancy between policy and practice in EI in Ghana, particularly in the provision of specialised education for deaf learners as previously reported by others [92,93,94]. To enhance EI for the deaf population in Ghana, there is a need for a well-defined policy on teacher training, encompassing pedagogies specific to deaf education, assessment techniques, audiological rehabilitation, and language and communication for deaf individuals. It is noted that a significant portion of teachers in inclusive settings appear to lack specialised training in deaf education [92,93,94]. Therefore, urgent action was recommended by the Ghanaian government to develop a competent human resource for quality EI delivery across the country [95]. In addition, the exclusive use of Ghanaian Sign Language (GhSL) for instruction and socialisation in deaf schools requires re-evaluation, suggesting the inclusion of a curriculum policy that offers the option of speech instruction for children interested and capable of learning spoken language. Moreover, GhSL was reported to lack full recognition in Ghana’s legal and policy frameworks, highlighting the need for reconsideration to officially acknowledge and incorporate GhSL as a subject in school language programs.

In another LMIC context, Ndegwa et al. [42] investigated EI Strategies post-hearing impairment diagnosis for children in Kenya. As Kenya was reported to be in the imminent position to roll out UNHS, and given Kenya’s structural issues, including compromised infrastructure, it is necessary that EI must be a component that follows early identification of hearing loss with appropriate referral pathways [42]. Appropriate supportive policy was described as imperative. Key EI strategies, with adequate policy as a central theme, that emanated from their joint Nairobi-Seattle group meeting are documented as follows:

- Establishing an Early Hearing Care (EHC) policy that covers early detection, intervention, habilitation, and family engagement.
- Developing a referral system that incorporates habilitation services and family engagement.
- Garnering support from the Ministry of Education and relevant organisations to facilitate EI and education.
- Expanding community and family support structures to enhance habilitation efforts and reduce stigma.

Of note, is the inclusion of multi-sector stakeholders drawn from academic institutions, the Ministry of Health, the Ministry of Education, the Council of Governors, hospitals, non-governmental organisations, and professional associations [42]. This underscores the complexity inherent in policy formulation applied into practice, with an upstream approach, where grass roots expertise is sought to ensure application feasibility. Such considerations are also required for the South African context.

In the South African context, Karisa et al. [96] sought to understand inclusive intervention service provision in ECD within the framework of nurturing care (NCF) and within the context of service migration from the DSD to DBE. Their theoretical article expounds on nurturing care as a significant framework supported by varying social contexts such as the home, child-care, the broader community, and policy influences. They elucidate that in order to achieve NCF ideals within the South African context, the following strategies should be applied: service localisation to account for cultural dynamics; developing strategies and tools for screening and EI that are contextually grounded, augmenting caregiver efficacy to facilitate improved childhood outcomes; staff training and support to facilitate improved child management and ECI, ECD stakeholder collaborations at a national, provincial, and local level, between government and non-governmental community-based organisations. Grass-roots involvement is touted as critical if poverty considerations that are common within the South African context are to be considered. As such, Karisa et al. [96] highlight the need to consider the engagement of families and communities of children with disabilities as essential. They further recommend that the Department of Basic Education (DBE), DSD, and DoH should consult directly with families and communities of children with disabilities such as hearing impairment to determine the most effective approaches for implementing ECI and ECD.

Naidoo and Kahn [63], in their South African study on barriers and facilitators to EHDI in the South African province of Kwazulu-Natal, highlighted the infrastructural issues already expounded under EHDI/EI Mechanisms within this review. What is of interest under this review’s Policy theme is Naidoo and Khan’s specific mention of the need to facilitate and strengthen intersectoral collaboration, where their participants highlighted poor private and public audiologist collaborations, and what was termed a disconnect between the DoH and the DBE. They added that mandating EHDI guidelines at a political level would facilitate a reduction in challenges.

## 4. Conclusions

As is evident from the above review, many factors need to be considered in the EI and EE policy implementation processes for HI/D/HH children. These processes include considerations of infrastructure, logistics, and population dynamics that affect policy formulation and implementation. EI and EE policy and guideline design and formulation are complex as well as nuanced and dependent upon the dictates of contexts within contexts. This is evident in both HIC and LMIC environments, where the heterogeneity of contexts at a geographical, infrastructural, economic, and population level, demonstrates sometimes extreme diversity in the same region. Most articles incorporated in this review included indirect consideration of policy as a function of EI timing, EHDI/EI mechanisms, EI-specific services rendered, family considerations, and EE transitioning, with nine being included under the Policy theme. However, only five out of the total deliberated policy aspects directly. This highlights the refined considerations often required for policy formulation, where fundamental societal workings as they interface with EI service provision require scrutiny. These include aspects such as poverty, economics, social, cultural, and linguistic diversity, which must be incorporated for effective policy implementation. South Africa, as a prime example of an LMIC context with its socio-cultural, economic, and linguistic diversity, has demonstrated gaps at the policy-practice junction. As such, South African policy needs to be reviewed with key stakeholder FCEI-DHH considerations, especially at the point of EI and EE service receipt. Further implications within the South African context relate to continuity of care, especially regarding transitioning between services such as health to education.

To attain advantageous outcomes for children with hearing impairment, beyond early detection, EI services are considered the point of initiation to attain the desired outcomes. For the HI/D/HH child aged six and below, particularly in LMIC contexts, astute scrutiny of current EI policies will more appropriately facilitate the establishment of contextually applicable and workable holistic, multi-speciality EI and EE mandates. Of particular importance is the inclusion of a bottom-up approach, where grass-roots service-recipient stakeholder perspectives must be included. Such steps will better facilitate the demarginalisation of HI/D/HH children in both HIC and LMIC contexts, which is an urgent imperative.

Current findings should take into account the identified limitation of the evidence timeframe. It is acknowledged that the timeframe of 2014 to 2024 for articles sourced, although appropriate, may have excluded sources that are relevant despite being somewhat outdated. In addition, although grey literature was justifiably excluded, publication bias may have increased as a result. Lastly, the article inclusion criteria were limited to English articles, which may have excluded relevant sources. Interpretation of results is thus cautioned with due consideration of these limitations.

## Data Availability

As an integrated review was conducted, data was extracted and synthesised from existing peer-reviewed articles accessed online. A summary is available via Supplement Table S1. As such, no new data were created.

## Supplementary Materials

The following table can be downloaded from: https://www.mdpi.com/article/10.3390/audiolres15010010/s1, Table S1: Integrative review: article-defined details (*n* = 26).
