# Supplementary material for: Early Intervention for Hearing-Impaired Children—From Policy to Practice: An Integrative Review
Source: Audiol Res. 2025 Jan 24;15(1):10. doi: 10.3390/audiolres15010010 (PMC11852310; doi:10.3390/audiolres15010010)
Supplement: Supplementary file 1 [file audiolres-15-00010-s001.zip › audiolres-3398508-supplementary.pdf]

Supplement 1 (S1): Integrative Review: Article Defined Details (*N*=26); Note: N/n herein refers to the number of studies included in the final sample)

**STUDY REFERENCE (*N*=26)**<br><br>**HIGH INCOME COUNTRY (HIC)**<br>***n*=13**	**Context & Location**	**Method**<br><br>• **Empirical/Theoretical**<br>• **N**<br>• **Design**<br>• **Objectives**	**Results Overview in Reference to Themes/Subthemes Derived**	**Recommendations**
1<br><br>Curle et al. (2017), [71]	(HIC)<br><br>Canada	• Empirical<br>• N=23<br>• Design: Qualitative<br>• Objective/s: Examine communication among early intervention (EI) providers, parents, and teachers during the transition to kindergarten for children who are deaf or hard of hearing.	• High-intensity transition support and fluid communication facilitated transition.<br>• Thorough information and resources from EI programs were helpful.<br>• EI professionals addressed parental stress and anxiety with emotional support and information and frequent school updates.<br>• Written reports from EI professionals to teachers of the deaf were crucial for preparing accommodations.<br>• Lack of routine communication pathways from schools to EI systems hindered knowledge transfer.<br><br>**THEME: Early Education (EE)**	• **Policy Updates**: EI programs updating about changes in school policies for kindergarten incoming deaf/hard of hearing (D/HH) children.<br>• **Communication**: Ensure teachers of the deaf frequently communicate with parents during the kindergarten year.<br>• **Parent Support**: Provide opportunities for parents of D/HH children to connect for support and information sharing.<br>• **Advocacy**: Enable parents to advocate for their children by voicing concerns about unsupportive practices.<br>• **Conflict Resolution:** Conflict resolution training by school districts and promote openness to parent perspectives.
2<br><br>Zaidman-Zait et al. (2019), [72]	HIC<br><br>Canada	• Empirical<br>• N=77<br>• Design: Mixed method.<br>• Objective/s: Describe parents' concerns, transition-related support practices, and explore perspectives of parents and teachers regarding practices that support a smooth transition to school for D/HH children.	• Communication between itinerant teacher of the D/HH with early interventionist/classroom teacher (67.57%)<br>• Teacher of the D/HH meeting and communicating with family prior to transition (59.46%)<br>• Determining school placement and needed supports (54.05%)<br>• Teacher of the D/HH observing the child prior to school entry (48.65%)<br>• Review of EI/audiological records (45.95%)<br>• Notification of D/HH student entering school system (43.24%)<br>• Facilitators of smooth transition:<br>○ EI program provision of informational support and direct guidance	• Future research on transitions to specialised settings, parent experiences, and geographical differences.<br>• Emphasis on the need for clear parent support, parent advocacy training for parents, intensive services for unique needs, and examining teacher demographics to understand their impact on transition practices.

			o Pretransition and ongoing communication between school personnel and family<br>o School planning for child's services and accommodations<br>o Communication between EI, medical, and school systems<br>o Unique Parent Themes:<br>- Family's personal and emotional resources<br>- Child characteristics<br><br>**THEME: EE**	
3<br><br>Awad et al. (2019), [43]	HIC<br><br>United States of America (USA)	• Empirical<br>• N-34<br>• Design: Quantitative<br>• Objective/s: To evaluate alignment with JCIH 1-3-6 guidelines and evaluate influencing variables.	• Hearing impairment diagnosis in 62% of infants by 3 months of age.<br>• Hearing aid fitment of 48% of infants by 4 months of age.<br>• Early intervention average enrolment age = 4.58 months.<br>• Hearing aid fitment within 1 month of age from hearing loss diagnosis for 70% of<br>• Average time of 1.68 months from diagnosis to hearing aid fitment<br>• Timely hearing aid fitting barriers: appointments missed/cancelled, middle ear involvement, and mild hearing loss.<br><br>**THEME: Early Intervention (EI) Timing**	• **Scheduling Adjustments:** Modify scheduling and appointments to meet JCIH guidelines for diagnosis by 3 months and hearing aid fitting within 1 month.<br>• **Examine Barriers:** Investigate why guidelines aren't met and find solutions.<br>• **Reduce Visits:** Decrease the number of visits for diagnosis and address family barriers to timely services.<br>• **Enhance Communication:** Improve communication with stakeholders and educate parents.
4<br><br>Alam et al. (2016), [39]	HIC<br><br>USA	• Empirical<br>• N=57<br>• Design: Quantitative<br>• Objective/s: To discuss reasons for non-standardisation in data reporting and to provide suggestions for improvement.<br>• Design: Quantitative	• In some jurisdictions, although progress evident, loss to follow-up/loss to documentation (LFU/LTD) rates remain high.<br>• Lack of data reporting standardisation impacts LFU/LTD.<br>• CDC survey updates have been made to improve standardisation of data.<br>• For improvement, several CDC strategies (3 EHDI-related quality measures, EHDI program staff education) developed and in place.<br><br>**THEME: EI Mechanisms –** ***Data management/systems***<br>**THEME: Policy**	• **Data Standardisation**: Data definitions and reporting methods updating for the Hearing Screening and Follow-up Survey (HSFS) to enhance consistency.<br>• **EHDI-IS Guidelines**: Creating standards to direct the technical and functional aspects of EHDI information systems.<br>• **Interoperability Efforts**: Promoting national initiatives to improve the integration between clinical and public health systems.<br>• **Quality Metrics**: Formulating standardised EHDI-related quality measures approved by the National Quality Forum.

				• **Ongoing Collaboration**: Maintaining continuous cooperation among the CDC, healthcare providers, stakeholders, and EHDI programs to bolster EHDI information systems.
5<br><br>Meinzen-Derr et al. (2022), [69]	HIC<br><br>USA	• Empirical<br>• N=1262<br>• Design: Quantitative<br>• Objective/s: To quantify the intensity of early intervention (EI) services allocated to 1262 children who were deaf or hard of hearing (D/HH) within a state program and to identify factors associated with intervention intensity.	• Majority of children (60.2%) received four or more EI services.<br>• 89% received service coordination in the first year; 60% received specialised D/HH services.<br>• Median service intensity at first IFSP: 73.3 minutes/month; increased to 121.2 minutes/month in the first year and 138.1 minutes/month across all years.<br>• Enrolling into EI by 6 months associated with lower intensity than enrolling after 12 months.<br>• Black/African American children received significantly lower EI intensity compared to white children.<br><br>**THEME: EI Services** – ***EI service intensity***	• Further research on service intensity and quality to refine EI services and ensure equitable access and outcomes.
6<br><br>Ward et al. (2019), [73]	HIC<br><br>USA	• Empirical<br>N=554 (surveys);<br>N=11 (focus groups)<br>• Design: Mixed methods<br>• Objectives: to ascertain the needs of families of young children who are deaf or hard of hearing (D/HH), and what supports, resources, and opportunities EHDI programs and EHDI-contracted Family-based Organisations (FBOs) were providing to families.	• Need for EHDI process information at its different stages.<br>• Importance of trusted, coordinated supports with resources.<br>• Value of inter-family support.<br>• Necessity for reliable, easily navigated and understood websites.<br>• Benefit of having a single contact person to align families with resources.<br><br>**THEME: Family Considerations**	• **Comprehensive Support:** Provide families with information on child development, communication options, and early intervention.<br>• **Awareness and Involvement:** Enhance family awareness and participation in EHDI programs through better outreach and website content.<br>• **Leadership Opportunities:** Increase family leadership roles by removing barriers.<br>• **Underserved Needs:** Address the needs of underserved families, including those in rural areas, minority groups, and those with children with additional disabilities.

7<br><br>Cole et al. (2019), [66]	HIC<br><br>USA	• Empirical<br>• N=161<br>• Design: Qualitative<br>• Objective/s: To investigate the perceptions of stakeholders working in Colorado's Part C system regarding the use of telehealth.	• Minimal telehealth utilisation despite its feasibility, allowing for more visits and sessions during non-traditional hours.<br>• Telehealth allows rural access to specialists.<br>• Telehealth models heighten family engagement and the use of coaching practices.<br>• Perception of telehealth as less effective and less personal<br>• Barriers: internet technology issues, negative family attitudes, lack of provider training in family coaching.<br><br>**THEME: EI Mechanisms – *Tele-Audiology***	• **Enhance Telehealth Training:** Increase training for providers to improve the delivery of telehealth services.<br>• **Address Internet Access:** Resolve internet access issues to ensure effective telehealth sessions.<br>• **Highlight Telehealth Advantages:** Emphasise telehealth's flexibility and increased family engagement.<br>• **Assess Telehealth Effectiveness:** Continue evaluating the effectiveness of telehealth compared to in-person visits.
8<br><br>Holzinger et al. (2021), [52]	HIC<br><br>Austria	• Empirical<br>• N=50<br>• Design: Quantitative<br>• Objective/s: To develop and implement a low-cost tracking system to follow infants from NHS failure, through ENT confirmatory diagnosis, to enrolment in a family-centred EI in Upper Austria.	• Proportion of children enrolled in early intervention before 6 months of age increased from 26% to 81%.<br>• Coverage for bilateral screening increased from 91.4% to 97.6%.<br>• Mean age at enrolment in early intervention reduced from 11.5 to 4.8 months.<br>• High number of children younger than 3 months of age at enrolment in early intervention (70%) compared to the previous cohort (4.3%).<br><br>**THEME: EI Mechanisms – *Data management/systems***	• **Multi-Professional Network**: Stakeholder network creation (maternity wards, paediatrics, ENT, early intervention providers) guided by health authorities for NHS program development and implementation.<br>• **Active Guidance and Quality Assurance:** Continuously guidance and quality assurance by health authorities by regularly collecting process indicators.<br>• **Adapt Documentation Models**: Tailor international documentation models to fit national/regional resources and legal conditions to facilitate cooperation.<br>• **Key Implementation Factors**: Limit screening repetitions, have maternity wards arrange ENT appointments, and ensure early intervention providers actively invite families for enrolment.
9<br><br>Folger et al. (2019), [53]	HIC<br><br>USA	• Empirical<br>• N=1,746<br>• Design: Quantitative<br>• Objective/s: To develop and characterise a population-based, longitudinal database documenting state-level services and outcomes	• Successful linkage of 1,262 records with EI data and 502 records with education data.<br>• Hearing loss confirmation median age 3.9 months<br>• Data linkage process challenges need for manual verification, absence of certain linkage variables.<br><br>**THEME: EI Mechanisms – *Data management/systems***	• **Model for EHDI Programs**: Integrated data systems across state agencies can serve as a model for other state EHDI and public health programs for children with disabilities to also enable future research on state EHDI outcomes.<br>• **Further research to** assess the impact of early intervention services on key outcomes.

		for children who are D/HH.		
10<br><br>Deng, Gaffney & Grosse (2020), [38]	HIC<br><br>USA	• Theoretical<br>• Reflection of state-level national efforts (data from state EHDI-IS and Hearing Screening Follow up Survey (HSFS)	• Higher screening rates in states with UNHS legislation<br>• Hearing screening conducted on over 95% of newborns before one month of age.<br>• Over 6,000 infants identified early each year.<br>• Less than 50% of infants of screening refers receive timely diagnosis; less than 50% enrolled in intervention by 6 months.<br>• Benchmark attainment vary markedly between states.<br>• Real-time decision support and data access in EHDI-IS systems.<br>• Geographic, racial, and socio-economic disparities affect service provision.<br><br>**THEME: EI Mechanisms – *Data management/systems***<br>**THEME: Policy**	• **Stakeholder Efforts:** Maintain ongoing efforts from all stakeholders.<br>• **Early Identification and Best Practices:** Public health agencies should emphasise early identification and support best practice implementation.<br>• **Surveillance Systems:** Enhance systems for population-based surveillance to improve tracking, follow-up, and reporting.<br>• **Specialised Services:** Boost efforts to make specialised services more accessible to underserved populations.
11<br><br>Curle et al. (2017), [40]	HIC<br><br>Canada	• Empirical<br>• N=10<br>• Design: Qualitative<br>• Objective/s: To investigate the organisational policies, procedures, and guidelines that facilitate or hinder the transition from EI to school for children who are D/HH from the perspective of program administrators.	• Facilitators:<br>○ Document exchange and interagency communication (80%)<br>○ Pre-transition school personnel child observation (70%)<br>○ Information and support provision to parents (60%)<br>○ Child and parents meet new school community (60%)<br>○ Key person to facilitate transition (50%)<br>• Barriers:<br>○ Stakeholder lack of or poor communication (70%)<br>○ Reduced time for adequate preparation (60%)<br>○ Reduced human and financial resources (60%)<br>○ Reduced placement options for D/HH (50%)<br>○ Stakeholders' reduced knowledge of D/HH student needs (50%)	• Future research to evaluate improve curricula and practice alignment between EI programs and school entry to assist school transition navigation for parents, children and teachers

			○ Reduced information/misinformation regarding school (50%)<br>• Wish List:<br>○ Clearer or simpler transition process (50%)<br>○ Stakeholder communication (40%)<br>○ More knowledge about D/HH needs among stakeholders (30%)<br>○ Strong supports to start school (30%)<br>○ More school resources (30%)<br><br>**THEME: Policy**	
12<br><br>Davenport & Weir (2022), [90]	HIC<br><br>USA	• Theoretical<br>• Objective/s: Proposed TEAM approach to assist in the transition process for D/HH children from EI to preschool.	• Early intervention (EI) services are crucial, with better outcomes for children enrolled by 6 months.<br>• Individualised Education Plans (IEPs) should consider language and communication needs.<br>• The TEAM approach involves transition planning, educational team establishment, accommodations, and connections.<br><br>**THEME: Policy**	• Transition planning engagement.<br>• Establishment of an educational team with professionals experienced in educating D/HH children Learning environment accommodations provision and ensuring communication access<br>• Collaborations for continued success.
13<br><br>Snoddon (2021), [91]	HIC<br><br>Canada	• Empirical<br>• N=3<br>• Design: Qualitative<br>• Objective/s: To analyse participant experiences in a daycare setting for a young deaf child who benefits from American Sign Language (ASL).	• Restricted resource access and policy barriers create marked gaps in public services for deaf children.<br>• Resultant parent out-of-pocket payments for ASL services<br>• Lack of training opportunities and qualified personnel and for deaf early childhood educators.<br>• Disintegration of previously integrated services.<br>• Policy requirement for parents to choose between sign language services and auditory visual therapy (AVT), rather than bilingualism support.<br>• The ASL consultant services provided often follow a rehabilitative model rather than an inclusive one.<br>• Reduction in flexibility and availability of needed supports due to policy changes in the Ministry of Education's preschool home visiting program.<br>• Despite these issues, participants still support inclusion.<br><br>**THEME: Policy**	• **American Sign Language (ASL) Training and Parental Support:** Mandate ASL training for early childhood educators and offer extensive ASL learning resources for parents of deaf children.<br>• **ASL-Fluent Daycares:** Create daycares staffed with ASL-fluent personnel to foster bilingual environments.<br>• **Individualise Healthcare Plan (IHP) Policy Adjustments:** Permit parents to select both spoken and sign language services.<br>• **Professional Development:** Enhance training and collaboration with deaf professionals in early childhood education and intervention.<br>• **Leadership and Policy Advocacy:** Strengthen leadership from daycare managers and policymakers to support these initiatives.

Supplement 1 (S1): Integrative Review: Article Defined Details (*N*=26); Note: N/n herein refers to the number of studies included in the final sample)

**STUDY REFERENCE**<br><br>**LOW-MIDDLE-INCOME COUNTRY (LMIC) – NON-SOUTH AFRICAN**<br><br>***n*=3**	**Context & Location**	**Method:**<br>• **Empirical/Theoretical**<br>• **N**<br>• **Design**<br>• **Objectives**	**Results Overview in Reference to Themes/Subthemes Derived**	**Recommendations**
1<br><br>Oppong et al. (2024), [41]	LMIC<br><br>Ghana	• Empirical<br>• N=24<br>• Design: Qualitative<br>• Objective/s: Examine policies and practices of inclusive education for young deaf children in Ghana; explore connections between teachers, clinicians, and caregivers in promoting early childhood care education (ECCE) for young deaf preschoolers.	• Parent education on basic sign language and provision of counselling through parent teacher association (PTA) meetings<br>• Challenges: parent weekend visits by parents, financial constraints, infrequent PTA meetings.<br>• Excursions and interactions with mainstream schools support social and communicative development.<br>• Early assessment and identification of hearing loss are critical for special schools' access.<br>• Gap between policy and practice in inclusive education, with a need for better teacher training and curriculum adaptation.<br>• Policies and laws do not fully recognise Ghanaian Sign Language (GhSL)<br><br>**THEME: Policy**	• **Inclusive Education Policy:** Develop clear policies for training teachers in deaf education.<br>• **Teacher Training:** accelerate training for qualified inclusive education teachers.<br>• **Language Options:** Reassess exclusive use of GhSL and add spoken language options.<br>• GhSL Recognition: Officially recognise GhSL as a school subject.
2.<br><br>Alyami et al. (2016), [44]	LMIC<br><br>Saudi Arabia	• Empirical<br>• N=60<br>• Design: Quantitative<br>• Objective/s: To determine the status of early intervention services provided to	• Children diagnosis at 13.7 months mean age.<br>• Hearing aids fitting at 20.7 months mean age.<br>• Intervention services commencement at 32.8 months mean age.<br>• Significant mean delay of 4 months between suspicion and diagnosis of hearing impairment)	• **Expand Services:** Extend newborn hearing screening and early intervention to all regions of Saudi Arabia.<br>• **Increase Access:** Boost the number of intervention centres and trained professionals, especially in rural areas.

		children who are deaf or hard of hearing and their parents/caregivers from birth to five years of age at two main state hospitals in Riyadh, Saudi Arabia, based on their parents' perceptions.	• Significant mean delay of 6.9 months between HI diagnosis and hearing aid fitting<br>• Earlier interventions received by those in Riyadh compared to outside Riyadh.<br>• Information regarding child's speech and language development needed by 96.7% of parents.<br><br>**THEME: EI Timing**	• **Remove Barriers**: Overcome obstacles like travel distances and lack of local services to ensure timely intervention for all children.
3.<br><br>Ndegwa et al. (2024), [42]	LMIC<br><br>Kenya	• Theoretical<br>• Objective/s: To discuss strategies for program development in Kenya and summarise key strategies for program strengthening identified during the workshop.	• Emphasis on the necessity of a national program providing support services for children with hearing loss.<br>• Importance of utilising existing government policies and enhancing collaboration between policymakers and local EHC advocates.<br>• Early Detection and Family-Centred Care stressed.<br><br>**THEME: Policy**	• **National Ear and Hearing Care (EHC) Program**: Comprehensive program development with support services involving families, communities, and healthcare workers, focusing on policy, early detection, intervention, and family-centred care.<br>• **Policy and Funding**: Update policies and secure funding to improve service accessibility, leveraging national healthcare, strengthening referrals, training the workforce, and setting up monitoring metrics.<br>• **Strategic Scale-Up**: Implement a phased scale-up in pilot counties with local policymakers' support, establishing EHC policy, early detection, intervention, and family engagement.
**STUDY REFERENCE**<br><br>**LMIC – SOUTH AFRICA (SA)**<br><br>***n*=10**	**Context & Location**	**Method:**<br>• **Empirical/Theoretical**<br>• **N**<br>• **Design**<br>• **Objectives**	**Results Overview in Reference to Themes/Subthemes Derived**	**Recommendations**
1	LMIC<br><br>SA	• Empirical<br>• N=32<br>• Design: Quantitative	• No uniform data management system nationally; no consistent shared system within sectors.	• Introduction in South Africa of an online data management system for EHDI programs to support integrated care, decision-making, and service evaluation, while addressing challenges

Moodley & Storbeck (2017), [54]		• Objective/s: To determine the type of data management systems in use in South Africa, their ability to allow cross-disciplinary sharing, and the challenges associated with implementing electronic or online database systems.	• 44% use paper-based systems; 27% use paper and computer-based systems; 25% use computer-based systems only; 6% use web-based systems.<br>• Limited data sharing within hospitals; very little sharing across hospitals or in the private sector.<br>• Challenges: limited electricity points, frequent electricity cuts, limited budget, lack of time and staff for data entry, and internet connection issues.Predominant use of paper-based systems, lack of data sharing capabilities, challenges specific to the developing country context (electricity and internet issues).<br><br>**THEME: EI Mechanisms –** ***Data management/systems***	like funding, staffing, and access to electricity and internet.
2<br><br>Maluleke (2024), [75]	LMIC<br><br>SA	• Theoretical<br>• Objective/s: To review the current state of EHDI programmes in South Africa, focusing on challenges and dimensions of access.	• Progress made but limited availability and affordability.<br>• Importance of acceptability and accommodation, involving caregivers, and ensuring linguistic and cultural congruence.<br><br>**THEME: Family Considerations**	• **Family-Centred Programs:** Implement linguistically and culturally appropriate FC-EHDI programs.<br>• **Multilingual EHDI:** To enhance caregiver participation, conduct programs in all official languages.<br>• **Training:** EHDI personnel education on language and cultural competence.<br>• **Community Collaboration:** Caregivers and community leaders' collaboration to address unique challenges and needs.
3<br><br>Karisa et al. (2022), [96]	LMIC<br><br>SA	• Theoretical<br>• Objective/s: To illuminate the provision of appropriate, inclusive services in early childhood development, including early childhood intervention, for children with disabilities in South Africa.	• EI strategies and development tools with service localisation.<br>• Professionals and care-givers efficacy enhancement.<br>• Nurturing care contexts inclusion of early childhood development (ECD) centres<br>• Nurturing Care Framework's five components: good health, adequate nutrition, responsive caregiving, security and safety, and opportunities for early learning.<br>• Teachers training in impairment-specific skills and adopting the Universal Design for Learning approach.<br>• Provision of support programs for professionals and collaborations promotion among stakeholders.	• Implement support programs for professionals to enhance their ability to assist children with disabilities.<br>• Provide urgent training for teachers on impairment-specific skills.<br>• Encourage staff to take charge of their professional growth and involve stakeholders to create supportive environments.<br>• Foster collaborations among stakeholders to develop synergistic relationships to benefit children with disabilities.

			• Community based nurturing care inclusion of home-based and Itinerant ECD programs with toy libraries.<br><br>**THEME: Policy**	
4<br><br>Khoza-Shangase (2019), [16]	LMIC<br><br>SA	• Empirical<br>• N=19<br>• Design: Qualitative<br>• Objective/s: To explore factors compromising early intervention service delivery to hearing-impaired children in South Africa, as expressed by their caregivers.	• Limited availability of appropriate schools and health care facilities (68%).<br>• Long distances between services and places of residence (53%).<br>• Significant costs linked to services (medical expenses, boarding school costs).<br>• Limited skills and knowledge of professionals and teachers (48%).<br>• Limited community awareness about hearing impairment and available services (68%).<br>• Long waiting lists for intervention (36%).<br>• Logistical barriers such as cost of consultations, transportation, and time off work.<br>• Caregiver-related issues like not knowing where to go for help<br><br>**THEME: EI Mechanisms –** ***Logistics and infrastructure***	• **Policy and Budget Planning:** Departments of Health, Basic Education, and Social Development should plan policies and budgets for children with sensory impairments support.<br>• **Human Resource Strategy:** Develop a strategy to prioritise children with hearing difficulties and emphasise early intervention benefits.<br>• **Awareness Campaigns:** As part of early childhood development initiatives, campaigns by the Department of Health should on risk factors, early identification, and available resources
5<br><br>Meyer, Swanepoel & Le Roux (2014), [45]	LMIC<br><br>SA	• Empirical<br>• N=253<br>• Design: Quantitative<br>• Objective/s: To describe the status of diagnostic and intervention services in the South African private health care sector	• Average age at diagnosis: 11 months<br>• Less than 20% of infants receive amplification before 6 months.<br>• 72% provide hearing aid fitting services to children aged 0-36 months.<br>• 94% provide some form of early intervention services.<br>• Early intervention services include parent counselling (80%), parent guidance (77%), speech-language intervention (50%), support group services (12%), and auditory training (6%)<br>• Less than a third (27%) diagnose hearing loss at the target age of 0-3 months.<br><br>**THEME: EI Timing**<br>**THEME: EI Services –** ***EI service type***	• **Newborn Hearing Screening:** Include systematic hospital-based hearing screening in birthing packages, with referrals to paediatric audiologists for timely assessment and management.<br>• **Specialised paediatric Testing:** Conduct diagnostic tests at specialised facilities with comprehensive tools and experienced audiologists.<br>• **EHDI Infrastructure:** Focus on expanding an integrated, accessible EHDI infrastructure to ensure prompt service access and maximise early intervention benefits.

6.<br><br>Maluleke et al. (2024), [57]	LMIC<br><br>SA	• Empirical<br>• N=9<br>• Design: Qualitative<br>• Objective/s: To describe caregivers' expectations of EHDI services and evaluate the success or failure of these services.	• EHDI services failed to meet caregiver EHDI expectations.<br>• Accessibility: EHDI services failed to meet EHDI accessibility expectations resulting from reported barriers e.g. long distances, high costs<br>• Only two children met age-appropriate acquisition of language expectations.<br>• Enrolment of only one child in a mainstream school.<br><br>**THEME: EI Mechanisms –** ***Logistics and Infrastructure***<br>**THEME: Family Considerations**	• **Context-Specific Strategies:** Create tailored solutions to ensure sustainable, effective and evidence-based EHDI services.<br>• **Caregiver Involvement:** Acknowledge caregivers as key contributors to successful EHDI services.<br>• **Integrated Services:** Implement comprehensive, interdisciplinary EHDI services that cater to South Africa's diverse linguistic and cultural needs.
7.<br><br>Naidoo & Khan (2022), [63]	LMIC<br><br>SA	• Empirical<br>• N=12<br>• Design: Qualitative<br>• Objective/s: To determine the barriers and facilitators to EHDI in KwaZulu-Natal as reported by audiologists/Speech therapists and Audiologists (A/STAs).	• Reduced private and public healthcare resources, staff shortages, reduced EHDI knowledge and awareness of EHDI.<br>• Contextual relevance (urban versus rural).<br>• Poor private and public audiologist collaboration<br>• Department of Health (DoH) and Department of Education (DBE) disconnect.<br><br>**THEME: EI Mechanisms –** ***EI logistics; EI infrastructure***<br>**THEME: Policy**	• **Resource Allocation:** Increase resources for EHDI implementation.<br>• **Education and Training:** Enhance education and training programs.<br>• **Culturally Relevant Practices:** Develop practices and protocols that are contextually relevant and culturally and linguistically diverse.<br>• **Further Research:** Conduct additional research to address clinical implications and limitations.
8<br><br>Maluleke et al. (2019), [34]	LMIC<br><br>SA	• Empirical<br>• N=8<br>• Design: Quantitative<br>• Objective/s: To describe communication and school readiness abilities of children with hearing impairment in EI preschools; determine ages at identification and initiation of EI services.	• HI identification ages from 7 months to 4 years and 1 month, mean of 2 years and 3 months.<br>• Amplification age from 1 year and 6 months to 4 years and 4 months, median of 2 years and 8 months.<br>• EI services initiation from 1 year and 5 months to 4 years and 2 months, mean of 2 years and 5 months.<br>• Only three participants attained age-appropriate school readiness abilities.<br><br>**THEME: EI Timing**<br>**THEME: Early Education**	• Systematically implement research findings into practice by the Departments of Health and Basic Education to achieve Early Hearing Detection and Intervention (EHDI) goals inclusive of school readiness outcomes.<br>• Conduct future research with a larger, representative sample across all nine provinces of South Africa to determine national EHDI outcomes

9<br><br>Khoza-Shangase & Michal (2014), [50]	LMIC<br><br>SA	• Empirical<br>• N=70<br>• Design: Quantitative<br>• Objective/s:<br>• Primary aim: To investigate audiological management protocols for the paediatric population.	• Mean age of hearing impairment identification 23.65 months,<br>• Amplification provided to 85.71% of children.<br>• Time lapse between identification and amplification mean 7.11 months.<br>• Age at introduction into aural rehabilitation: Mean 2 years 5 months.<br>• Mode of communication:<br>• Auditory Verbal Therapy (AVT): 48.57%<br>• Sign Language: 18.57%<br>• Total Communication: 11.43%<br>• No Aural Rehabilitation: 14.29%<br><br>**THEME: EI Timing**<br>**THEME: EI Services** – ***EI service type***	• Mandate structured newborn hearing screening in Gauteng hospitals for early hearing loss identification.<br>• Tackle poor follow-up to ensure children benefit from provided services.<br>• Establish follow-up services for timely amplification and ongoing intervention for children with hearing loss.<br>• Audiologists to advocate for early hearing detection and intervention to reduce the impact of unidentified hearing loss.
10.<br><br>Störbeck (2024), [67]	LMIC<br><br>SA	• Theoretical<br>• Design: Qualitative<br>• Objectives/s: To advocate for specialised early childhood intervention tailored to young children with disabilities and their families, emphasising Family-Centred Early Childhood Intervention (Fc-ECI).	• Specialised intervention for children with disabilities recognised as an urgent need.<br>• Importance of focussing on empowering parents and individualised support provision.<br>• Importance of accessible ECD environments with inclusive early childhood development (IECD)<br>• Despite overall increase in funding for ECD, disparity for disability-focused programs remain<br><br>**THEME: EI Services** – ***EI service type***<br>**THEME: Family Considerations**	Governments should:<br>• Prioritise inclusive early childhood development for children with disabilities.<br>• Ensure adequate funding, resources, expertise, and time.<br>• Emphasise strategic, culturally sensitive implementation, public commitments, and accountability.<br>• Call for greater investment and prioritisation of inclusive early childhood development initiatives.<br>• Uphold human rights, providing equal access to nurturing environments, early education, and participation in cultural social and cultural activities.
